# Book Review: The Gay Revolution: The Story of the Struggle

**DOI:** 10.3389/fpsyg.2021.677734

**Published:** 2021-05-17

**Authors:** Alex Siu Wing Chan

**Affiliations:** Department of Applied Social Sciences, The Hong Kong Polytechnic University, Kowloon, Hong Kong

**Keywords:** homosexual rights movement, social inclusion & exclusion, civil rights movement, sexual minorities, LGBTQ

The Gay Revolution dates back to the 1950s. At that time, homosexuals were regarded as offenders. They were mentally impaired in the eyes of mental health professionals and wicked in the eyes of religious institutions, and the community was harassing them. The media stigmatizes homosexuals in relation to the judicial system, the armed forces, education, and the clinical profession. In this oppressive environment, several bold individuals tried to strike back, setting the stage for the progressive reforms in the 1960s and many years to come. What Faderman examines includes the movements of the 1960s, the resistance in the following two decades, the destabilized yet cohesive society after the AIDS crisis, and the existing barriers to the transition to marital fairness. Given that the magnate of transformation that Lesbian, gay, bisexual, and transgender (LGBT) individuals have been incredible, it is worthwhile to delve deeply into these events.

The Gay Revolution is an authoritative study of America's homosexual movement. It offers a comprehensive account of the contemporary movement for gay, lesbian, and trans rights, from the 1950s to the current time, focusing on fascinating conversations with political leaders, soldiers, civil advocates, and representatives of the LGBT population who experience such struggles on a daily basis. Centered on extensive analysis and over 150 interviews, the book reveals this ongoing tale not through plain details but through vivid descriptions of compelling battles. To bring us this thoroughly written publication, Faderman scoured the database of libraries, journals, governmental agencies, lesbian and gay groups, and arrest reports, as well as consulting over 100 campaigners.

Faderman's work discusses gay culture from a fresh and dynamic perspective. Maybe the greatest achievement is due consideration given to women. Faderman is frequently referred to as a “lesbian historian” because of her outstanding efforts in the area. She gathered more than 150 conversations for her latest work, including some that belonged many years ago, such as her conversation with the lesbian activists Del Martin and Phyllis Lyon in the late 1980s.

Faderman's dedication to gender equality is apparent at an initial point as a section on the masculine Mattachine Society is part of the Daughters of Bilitis (Chapter 6). The members from the headquarters of lesbian civil and political rights learned about their rights and gay culture from the DOB (p. 77). “Its very establishment despite police persecution was an act of bravery,” Faderman said, “because members still had to fear being attacked, not for what they did, but simply for who they were” (p. 78). As the book progresses, Faderman explains how Martin criticized her listeners at the 1959 Mattachine conference for seeing gay rights as something only for men. However, in 1970, Betty Friedan from the National Women's Organization voiced fear that, like Faderman described, lesbians might “give feminism a bad name,” labeling these individuals as Lavender Menace. In the “intersectionality” crisis, a term coined by identity intellectuals, lesbians were caught between two camps—the masculine homosexual community and the anti-lesbian feminist campaign (p. 89–90, p. 236–237).

Faderman's commitment to women is more widely a reflection of her balanced philosophy. That is a widespread accusation that the campaign has inadequately registered its conscripts. “The Gay Revolution” features the main villains and heroes of the movement—the accounts of Anita Bryant and Harvey Milk are especially noticeable (p. 333–334)—yet there is also an uplifting narrative highlighting the achievements of less well-recognized individuals, be they heterosexual or homosexual (chapter 7 & chapter 18–20). In 1953, psychiatrist Evelyn Hooker demonstrated to her coworkers that they could not tell the difference between the psychiatric evaluation findings of straight and gay people, thus starting to question the consensus of homosexuality as a medical group (p. 99).

Jeanne Manford had two gay sons—one died of opioid addiction and another was violently assaulted. After these events, she cofounded Parents and Friends of Lesbians and Gays, changing the entire paradigm by turning a gay outsider into a relative. Leonard Matlovich also laid the foundation for the public service of Gen. Tammy Smith when he protested the prohibition of gays by the Air Force in 1975. As with every description, this one has imperfections. Although the name L.G.B.T. is often used, the exclusion of transsexual people in this account is quite indicative of “L.G.B.T.” narratives. Then on the other end, there have been occasions where the wide variety of voices seeks to develop into a deafening roar. We leap from episode to episode quite rapidly. However, persistence is compensated as a complete understanding of the story is gained. Each stage of the movement is drenched in minute details such as individual experiences, something that is missing from other existing works.

Institutions as well as their efforts are being driven to the edges of Faderman's report (chapter 7). For years, the biggest LGBT institution in America has been the Metropolitan Community Church, which has religious communities throughout the country (p. 102–103). MCC is a Christian organization formed in 1968 with the aim of offering a supportive and validating venue for worship for LGBTQ and heterosexual church goers. The organization's Human Rights Protocol outlines the Christian obligation to “stand in solidarity with those who are marginalized and oppressed,” and to “lift up new generations of remarkable, far-reaching spiritual activists” (Stone, 2018). However, Faderman pays no heed to MCC. Institutions are the backbone of a particular cause, yet they are almost ignored in this report. More importantly, Faderman scarcely demonstrates two of the most dramatic ways of transition that have taken place over the past four decades. Because of gay demonstrations, many of which were initiated by LGBTQ African-Americans and therefore were closely associated with the civil rights movement, police brutality decreased significantly in the 1970s (Kane, 2013; Stone, 2016). That, in essence, facilitated the development of LGBT business areas and the political strength which might arise from it. Moreover, one might fairly say that Ellen DeGeneres and her “coming out” in the 1996–97 season of the show achieved even more to transform American people's perceptions than any of the others Faderman mentions in the narrative. Nevertheless, only one paragraph is devoted to the importance of DeGeneres. However, it is indeed reasonable that Faderman did not address the above since the objective of the book is to include individual accounts of marginalized people. Furthermore, no narrative is a flawless depiction of the development of a social justice campaign, and the immense clarity offered by The Gay Revolution outweighs these shortcomings.

Ignoring these events has made the book somewhat less comprehensive. By not considering such aspects in the context of the past 50 years, Faderman is often forced to use terms like “miraculous” and “fate” to describe what has happened. Obviously, some significant events are missing from the book. There is no discussion of the efforts of Midwestern or Southern advocates, and the omission of locations such as Chicago, Atlanta, and Miami, which are known for their exceedingly powerful campaigns, is an obvious issue. Despite these limitations, Faderman provides a credible analysis of a hidden portion of the past of the U.S., and of people with both frustrating and empowering experiences.

“The Gay Revolution” is a narrative about how a marginalized group has continued to struggle for their own interests notwithstanding enormous barriers. As a matter of fact, considering that LGBTQ people are becoming an increasingly committed, recognized, and visible part of society (Chan, 2021), “The Gay Revolution” is an exceedingly important book since it illustrates the origins and influences of the LGBTQ movement. I greatly appreciate it because it promotes the notion of social inclusion in today's world.

## Author Contributions

The author confirms being the sole contributor of this work and has approved it for publication.

## Conflict of Interest

The author declares that the research was conducted in the absence of any commercial or financial relationships that could be construed as a potential conflict of interest.

## References

[B1] Chan A. S. W. (2021). Book review: the deviant's war: the homosexual vs. the United States of America. Front. Sociol. 6:667576. 10.3389/fsoc.2021.667576

[B2] Kane M. D. (2013). LGBT religious activism: predicting state variations in the number of Metropolitan Community Churches, 1974–2000. Sociol Forum 28, 135–158. 10.1111/socf.12006

[B3] Stone A. L. (2016). The impact of anti-gay politics on the LGBTQ movement. Sociol Compass 10, 459–467. 10.1111/soc4.12373

[B4] Stone A. L. (2018). The geography of research on LGBTQ life: why sociologists should study the south, rural queers, and ordinary cities. Sociol Compass 12:e12638. 10.1111/soc4.12638

